# Cross-Subject EEG Feature Selection for Emotion Recognition Using Transfer Recursive Feature Elimination

**DOI:** 10.3389/fnbot.2017.00019

**Published:** 2017-04-10

**Authors:** Zhong Yin, Yongxiong Wang, Li Liu, Wei Zhang, Jianhua Zhang

**Affiliations:** ^1^Shanghai Key Lab of Modern Optical System, Engineering Research Center of Optical Instrument and System, Ministry of Education, University of Shanghai for Science and TechnologyShanghai, China; ^2^Department of Automation, East China University of Science and TechnologyShanghai, China

**Keywords:** emotion recognition, affective computing, physiological signals, recursive feature elimination, EEG

## Abstract

Using machine-learning methodologies to analyze EEG signals becomes increasingly attractive for recognizing human emotions because of the objectivity of physiological data and the capability of the learning principles on modeling emotion classifiers from heterogeneous features. However, the conventional subject-specific classifiers may induce additional burdens to each subject for preparing multiple-session EEG data as training sets. To this end, we developed a new EEG feature selection approach, transfer recursive feature elimination (T-RFE), to determine a set of the most robust EEG indicators with stable geometrical distribution across a group of training subjects and a specific testing subject. A validating set is introduced to independently determine the optimal hyper-parameter and the feature ranking of the T-RFE model aiming at controlling the overfitting. The effectiveness of the T-RFE algorithm for such cross-subject emotion classification paradigm has been validated by DEAP database. With a linear least square support vector machine classifier implemented, the performance of the T-RFE is compared against several conventional feature selection schemes and the statistical significant improvement has been found. The classification rate and *F*-score achieve 0.7867, 0.7526, 0.7875, and 0.8077 for arousal and valence dimensions, respectively, and outperform several recent reported works on the same database. In the end, the T-RFE based classifier is compared against two subject-generic classifiers in the literature. The investigation of the computational time for all classifiers indicates the accuracy improvement of the T-RFE is at the cost of the longer training time.

## Introduction

To improve the satisfaction level and the reliability of the human agents who interact or collaborate with machines and robots, intelligent human-machine (HM) systems with the capability of accurately understanding human communications are inevitably required (Soleymani et al., 2012). Since the human intentions and commands may carry various emotions in a verbal or a non-verbal manner, the proper response to the human affective behaviors is essential to achieve the self-adaptation of the machine and computers (Zeng et al., 2009; Fanelli et al., 2010). Considering most of the contemporary HM systems being unable to recognize emotional clues, emotion classifiers are developed, and aimed to provide temporal predictions of certain emotional states based on the integration of human reactions from facial/vocal expressions and/or physiological signals (Hanjalic and Xu, 2005; Kim and Andre, 2008).

The output of the emotion classifier can be determined by self-assessment techniques and the valence-arousal (VA) model. The VA model facilitates analyzing complex emotions based on the cores of affections (Russell, 1980; Lang, 1995). More specifically, different emotional states are labeled as points in a 2-dimensional space with each axis defined by arousal or valence degree so that the emotion categories can be visualized by the locations in the plane. Note that the VA model is closely associated with the limbic system that regulates emotions, long-term memories and behaviors (Zhang and Lee, 2013). In particular, the emotional responses reflected by the valence dimension are related to the activities of cortical networks under the insular cortex and the anterior parietal cortex (Anders et al., 2004) while the arousal dimension is associated with the activities in the right supramarginal gyrus (Zhang and Lee, 2013). In particular, the useful information from the continuous measurement of cortical activities can be extracted and selected to indicate the variations of the human cognitive sate. In recent study, Naseer et al. (2016) built a novel brain-computer interface system, where the linear discrimination analysis model is used to classify the functional near-infrared spectroscopy (fNIR) features. Based on the optimal feature combination, the optimal recognition rate of two mental states is achieved.

Since the human affective responses are linked to the cortical activities, electrophysiological measures of the central nervous system can be used as the inputs of the emotion classifiers. Among them, electroencephalogram (EEG) received much attention because of its high repeatability with low-cost, portable implementations (Birbaumer, 2006; Kim and Andre, 2008; Brunner et al., 2011). In well-documented works, the accessibility of EEG for estimating emotional states was extensively explored. Verma and Tiwary (2014) reported the EEG power spectral density features within alpha (8–13 Hz) band are associated with different valence levels. Balconi and Mazza (2009) reported the phase synchronization between the right and left scalp EEG could reflect the variations of the arousal levels. Konstantinidis et al. (2012). reported EEG power features of theta (4–7 Hz) band extracted from Cz, Fz, and Pz channels can indicate both arousal and valence levels.

To facilitate analyzing huge-volume, high-dimensional EEG data, the machine learning based estimators, and feature selection methods show the effectiveness on the issue of subject-specific emotion recognition, where a new classifier for each subject is built. Under such paradigm, Zhang et al. (2016) extracted EEG features for binary emotion classification by combining the empirical mode decomposition and sample entropy methods. Atkinson and Campos (2016) employed mutual information minimization technique and one-against-one support vector machines (SVMs) as the EEG based emotion classifier. Khezri et al. (2015) used three-channel forehead EEG combined with blood volume pressure and skin resistance to recognize six basic emotions via SVM and *k*-nearest neighbors (KNN) classifiers. Since very-high classification accuracies were found in above works, the subject-specific feature extraction and classification approaches are competitive when sufficient EEG training instances are available for a single user. However, it leads to a disadvantage that induces additional burdens to each subject and require long time for preparing multiple-session EEG data for reliably training classifiers since the EEG signal is known to be non-stationary and differently distributed in different days (Christensen et al., 2012; Zhang and Lee, 2013; Li et al., 2016).

To overcome the shortcoming of the subject-specific paradigm for emotion recognition, a promising solution is further generalizing the localized affective model trained on one subject to adapt for a novel subject. In recent studies on mental workload assessment issue, Wang et al. (2012) proposed a cross-subject hierarchical Bayesian classifier (CHB) to achieve the EEG based workload recognition. In their work, the classifier was trained and tested on the EEG features extracted from eight subjects. The classification performance of the CHB classifier is stable when three levels of workload were estimated. The accuracy of the CHB is also comparable to a subject-specific classifier. In addition, Baldwin and Penaranda (2012) proposed an adaptively trained artificial neural network (ATNN) to recognize operator workload by using EEG features. The ATNN classifier show stable classification accuracy across a group of subjects that operates different human-machine tasks. The above works focus attention on the classifier design with all possible EEG features employed. However, the robust EEG features from a wide variety of individuals are also important for subject-generic emotion classifier. A basic strategy is to select salient EEG features by the mixed data from all training subjects together (Yin and Zhang, 2014). This is usually infeasible since the data distributions between testing and training subjects are different. Hence, it is more practical to derive an EEG feature subspace that represents the training and testing data in similar modalities across historical data from a group of subjects and a novel subject. To this end, we attempt to develop a new EEG feature selection approach, transfer recursive feature elimination (T-RFE), to determining a set of the most robust EEG indicators with stable geometrical distribution across a group of training subjects and the specific testing subject who involved in the affective HM systems.

The proposed T-RFE algorithm is aimed to build the subject-generic emotion classifier. For instance, an EEG dataset of multiple subjects is available. For a novel subject used for testing, the conventional subject-specific classifier require a comprehensive training set building by multiple-session EEG data collected from the same subject. On the other hand, the subject-generic classifier can exploit the historical data from other subjects in the dataset. In such case, it is not necessary to collect long-time EEG recordings from the testing subject compared to the subject-specific classifier without the T-RFE based feature selection.

The motivation of the study includes two aspects: (1) the accuracy of the cross-subject, EEG-based emotion classifier is still limited because of the heterogeneity and individual-specificity in EEG time or frequency domain features and (2) the reliable cross-subject emotion recognition depends much on the proper selection of the EEG features that has the shared information in multiple individuals.

The standard RFE algorithm was developed based on the SVM in which the loss of the classification margin was used as objective function to evaluate the discriminative contributions of each feature (Vapnik, 2000; Guyon et al., 2002). When the feature possessing the lowest contribution is iteratively eliminated from the training set, it elicits rankings ordering the salient to non-salient features. Regarding to the fact that the cross-subject emotion recognition is a typical domain adaptation problem in transfer learning (Bishop, 2006), it is natural to generalize the conventional RFE to the T-RFE aiming at transferring common knowledge across two or more different subjects in a shared low-dimensional feature space. To explore the effectiveness of the T-RFE algorithm for cross-subject emotional feature selection, the EEG data from the public DEAP database of 32 participants were used. The T-RFE is also compared against the standard RFE and combined with the linear classifier to show its effectiveness on improving the accuracy of the cross-subject emotion recognition.

The rest of the paper is organized as follows. A short description of the DEAP database, EEG preprocessing, feature extraction, the methodology, and algorithms of the T-RFE are given in Section Backgrounds and Methods. Section Results provides the detailed results for feature selection, cross-subject emotion classification on arousal, and valence dimensions. The classification performance comparison is performed via non-parametrical statistical test. Some useful discussions on the properties of the T-RFE as well as the potential limitations of the present work are given in Section Discussions. A short conclusion of the contributions of the study is presented in Section Conclusions.

## Backgrounds and methods

### Data acquisition and splits

The DEAP database was used to evaluate the T-RFE feature selection algorithm for cross-subject emotion classification. Koelstra et al. (2012) built the database of 32 healthy subjects (19–37 years, mean = 26.9, 50% females) and made it publicly available for exploring human emotion variations induced by musical videos. During the data acquisition stage, each participant performed 40 trials of the experiments. For each trial, a video clip lasting 1 min was presented to the subject and the physiological data were simultaneously recorded. In total, the duration of the data acquisition of each trial lasted 63 s. It includes 3 s baseline condition and 60 s for participant watching the video. In the end of the trial, the participant was instructed to accomplish the self-assessment on valence, arousal, dominance, and liking scales from 1 to 9, where 1 and 9 indicate the lowest and the highest levels of each affective dimension, respectively.

**Figure 1:**
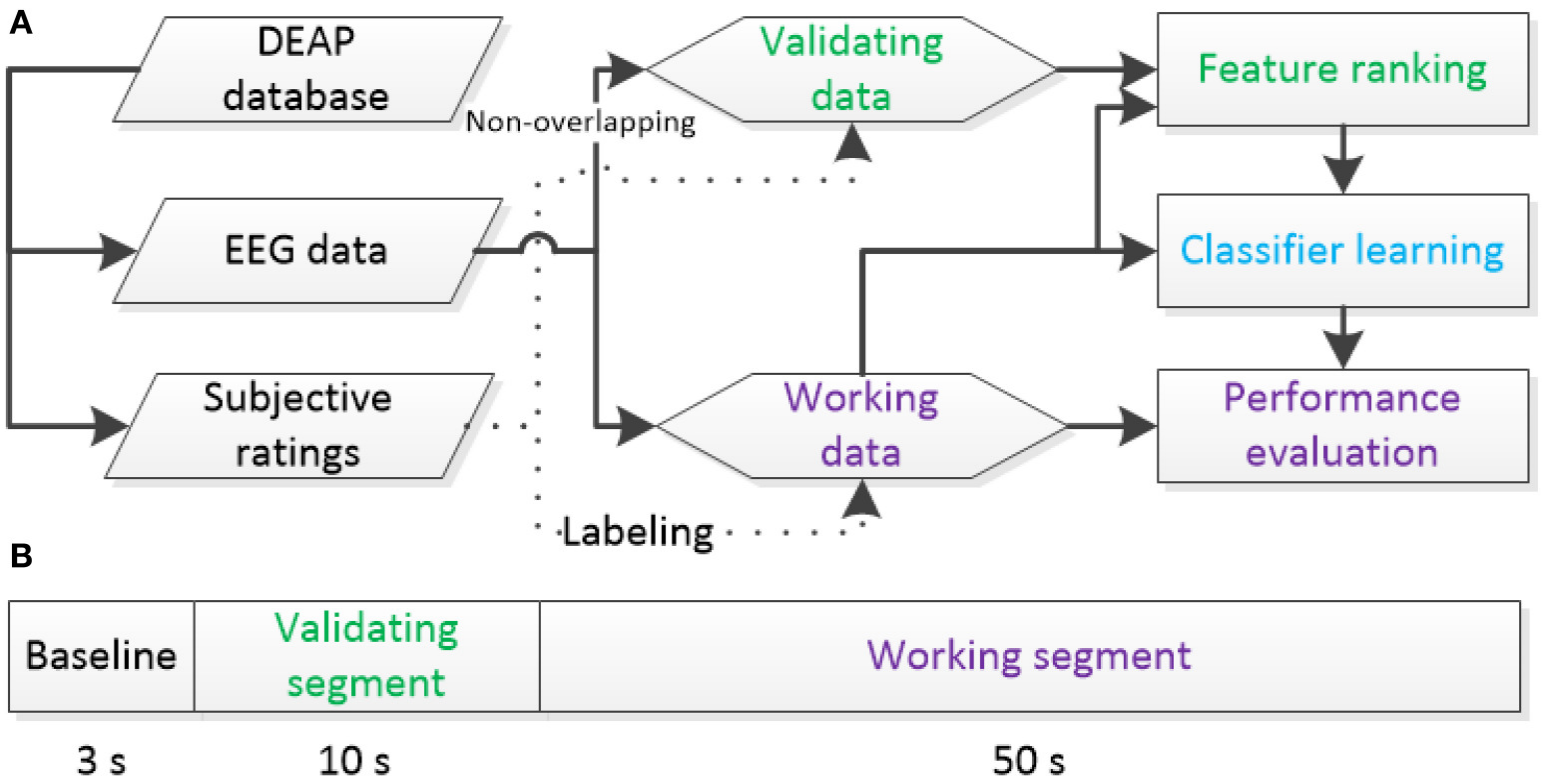
**Definitions of validating and working data sets from the DEAP database: (A)** the function of each set, **(B)** data splits from a trial to build two sets.

In this work, the VA model is used to determine the target classes of the emotions with liking and dominance scales excluded. For physiological data, the 60-s-length EEG signals of 32 channels sampled at 128 Hz are adopted for feature extraction without the baseline conditions. To avoid potential overfitting in T-RFE model selection, we use the first 10 s EEG signals of each trial to build a validating dataset for feature ranking. The remaining 50 s EEG signals are defined as a working dataset for feature selection, training, and testing the classifier. The functionalities of the two non-overlapping sets are shown in Figure 1. Note that the signal length of the validating data is smaller than that of the working data. The scheme in the figure is aimed to simulate a pseudo online classification environment with limited subject-specific EEG data available for ranking features.

### EEG data preprocessing and feature extraction

**Figure 2:**
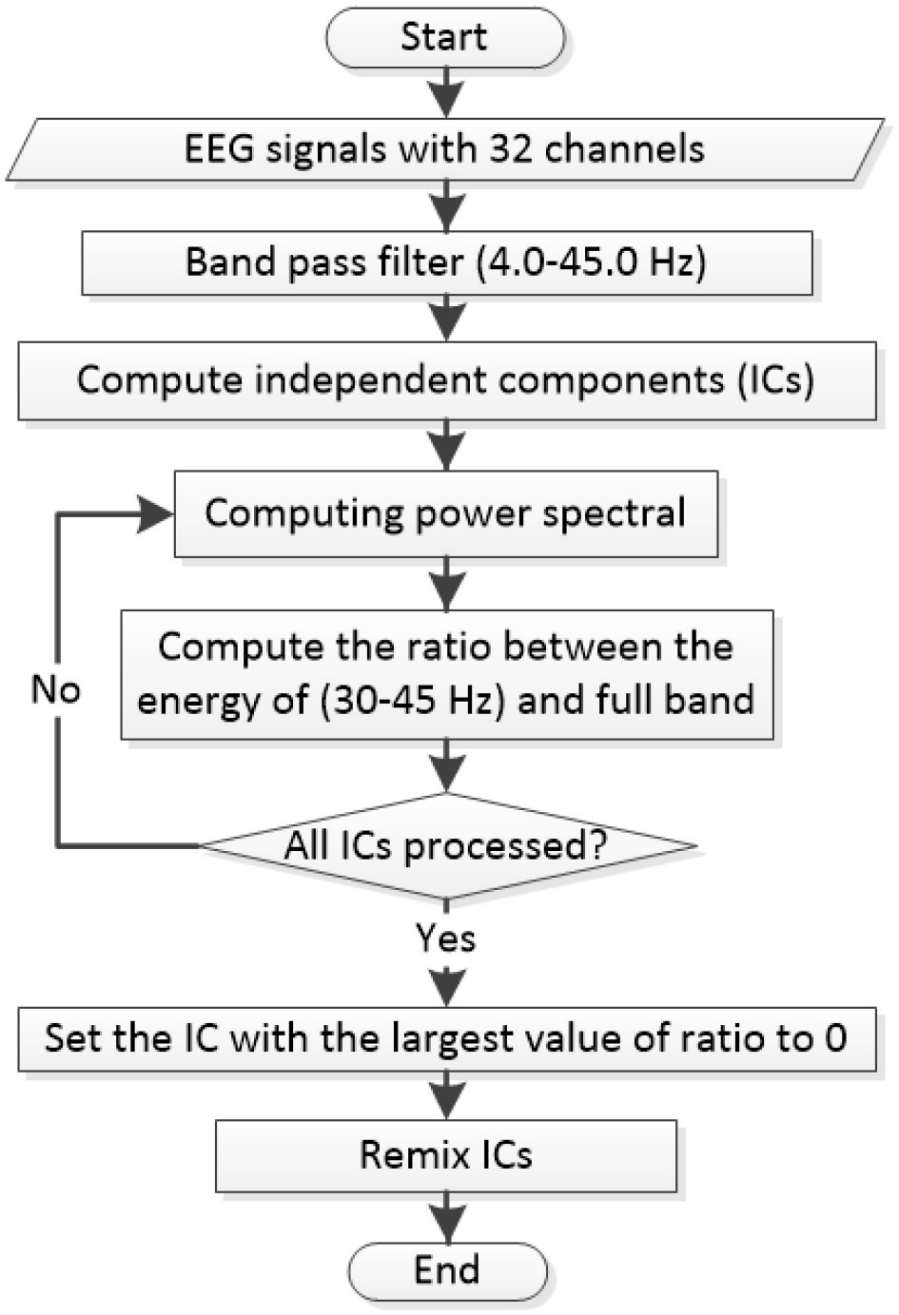
**Flowchart for EEG pre-processing by using the band-pass filter and the ICA transformation for each trial**.

All 32 channels of EEG signals in each experimental trial are preprocessed based on the flowchart shown in Figure 2. Note that the EEG preprocessing is applied before the data splits. A 3-order band-pass Butterworth filter with the cutoff frequencies of 4.0 and 45.0 Hz is first used to remove the unwanted noises originated by respiration and eye movements. Then, the filtered EEG data is processed via independent component analysis (ICA) to eliminate the myoelectric noise from scalp muscles.

**Figure 3:**
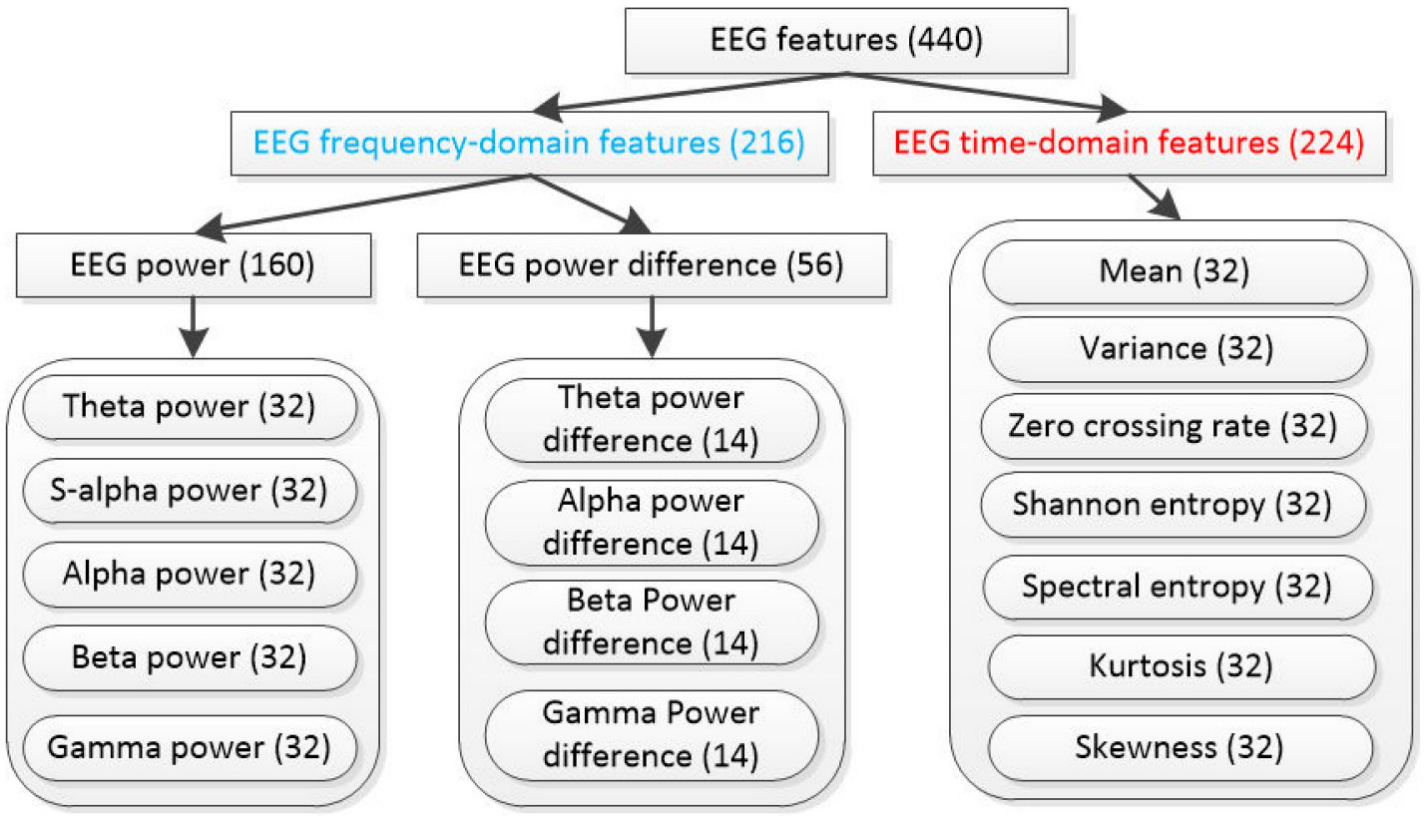
**Extracted EEG features from each data segment, the number in the parenthesis denotes the dimensionality of each feature type and s-alpha denotes “slow alpha” frequency band**.

After all EEG signals are preprocessed according to Figure 2. We divide the last 60 s EEG data of each trial into validating segment and training/testing segment based on Figure 1. For each segment, EEG features are extracted with 440 dimensions. The notations of the computed EEG features are summarized in Figure 3. In total, 216 frequency domain features are derived by computing the EEG power via fast Fourier transformation. For all channels (Fp1, AF3, F3, F7, FC5, FC1, C3, T7, CP5, CP1, P3, P7, PO3, O1, Oz, Pz, Fp2, AF4, Fz, F4, F8, FC6, FC2, Cz, C4, T8, CP6, CP2, P4, P8, PO4, and O2), 160 power features of five frequency bands [theta (4–8 Hz), slow-alpha (8–10 Hz), alpha (8–12 Hz), beta (12–30 Hz), and gamma (30–45 Hz)] are computed. We also extract 56 features of power differences between right and left cortical areas according to the reported work (Koelstra et al., 2012). The 14 channel pairs are employed (Fp2-Fp1, AF4-AF3, F4-F3, F8-F7, FC6-FC5, FC2-FC1, C4-C3, T8-T7, CP6-CP5, CP2-CP1, P4-P3, P8-P7, PO4-PO3, and O2-O1) while the power differences in four frequency bands (i.e., slow-alpha is excluded) are computed for each channel pair. In addition, seven EEG time-domain features (mean, variance, zero-crossing rate, Shannon entropy, spectral entropy, kurtosis, and skewness) are computed for each channel. To eliminate feature scale differences, we separately standardize features among 40 trials of each subject into mean = 0 and *s.d*. = 1.

Each iterative of the T-RFE feature selection requires both of EEG data from a source domain and a target domain. For a specific testing subject, the remaining subjects provide the EEG data of the source domain. A small among historical data from the testing subject build the target domain dataset, i.e., the first 1/6 trial signal of the testing subject. In the end, the remaining 5/6 trial data of the testing subject is used to investigate the classifier performance. Therefore, the feature selection model is predetermined only based on the training and validating data without any testing data. That is, the overfitting of the T-RFE model can be avoided since it performances are independently evaluated. In addition, the linear LSSVM is used to build the T-RFE and the parsimonious structure of the linear model can naturally avoid the overfitting issue.

### Target emotion classes determination and classification performance evaluation

Both of the feature selection and the classifier design are based on the supervised learning methodologies. Hence, the target emotion class (or the ground truth) of each feature vector must be predetermined before applying feature selection and classification. In order to quantitatively measure, the valence and arousal degrees for each participant, the technique of the self-assessment manikins (Koelstra et al., 2012) were used. That is, there are nine manikins with different expressions displayed in the computer screen with the numbers 1–9. The emotions can be easily indicated based on the manikin expressions. Participants are instructed to move the mouse horizontally below the numbers and clicked to indicate the arousal and valence scales. More specifically, 1 and 9 indicate the lowest and the highest degree of arousal or valence scales, respectively. In recent reported works on DEAP database (Koelstra et al., 2012; Atkinson and Campos, 2016), the binary emotional classes are usually generated based on a fixed threshold, e.g., five and the rating data are directly discretized into low (< 5) and high (≥5) arousal (or valence) states. However, since the subjective ratings also possess the non-stationarity and subject-specificity (Zhang et al., 2015), the fixed threshold may not be suitable for all individual preferences on the video clips. Hence, a personal threshold generating subject-specific emotional classes with self-assessment personalities could be much proper. Motivated by this, we determine the target classes by clustering subjective rating data for each subject, where the threshold is computed by the midpoint of two cluster centers. The classical *k*-means clustering algorithm is repeatedly applied on 40 observations of subjective rating data of a subject, i.e., {**z**_1_, **z**_2_, …, **z**_40_}, ${\text{z}}_{i}\in R^{2}$. Two entries in **z**_*i*_ denote the values of the valence and arousal dimensions. The cluster centers {**c**_1_, **c**_2_} are elicited by,

(1) $$\begin{array}{r} {\text{c}}_{j}=\arg\min g\left({\text{c}}_{j}\right)=\arg\min\overset{2}{\underset{j=1}{\sum }}\underset{{\text{z}}_{i}\in C_{j}}{\sum }{\left||{\text{z}}_{i}-{\text{c}}_{j}|\right|}^{2}. \end{array}$$

Then, the 2-D coordinates {τ_1_, τ_2_} of the midpoint **τ** of two cluster centers are the adaptive thresholds for valence and arousal dimensions,

(2) $$\begin{array}{r} \tau =\left\{\tau_{1},\tau_{2}\right\}=\frac{1}{2}\overset{2}{\underset{j=1}{\sum }}{\text{c}}_{j}. \end{array}$$

**Figure 4:**
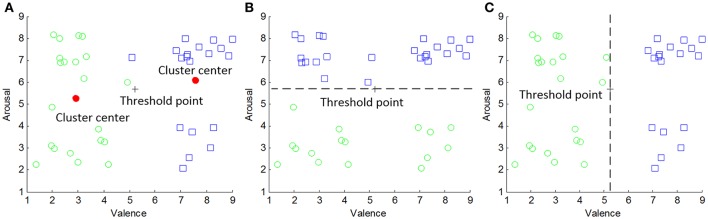
**Determination of adaptive thresholds and target emotion classes for subject 1: (A)** results of *k*-means clustering, **(B)** target classes for arousal dimension, and **(C)** target classes for valence dimension.

An example of how to determine the personal thresholds and corresponding target emotion classes for subject 1 are shown in Figure 4. In Figure 4A, two clusters of the subjective rating data marked by circles and squares are elicited. Two dots in the VA plane are the cluster centers. The cross marker represents the center midpoint with {τ_1_, τ_2_} = {5.2342, 5.6803}. That is, the low and high arousal states can be discretized by the threshold of 5.6803 (see Figure 4C) while that of 5.2342 defines the same binary valence states (see Figure 4B). It is shown the unsupervised clustering successfully learns the information for two preferences of rating for a single subject while the cluster interpretation can be achieved by examining cluster centers. Table 1 summarized different threshold values for 32 subjects. From the table, the threshold varies across all individuals but is close to the classical fixed value of 5. Thus, the subject personality can be reflected by the slight variations, e.g., 0.6803 and 0.2342 for arousal and valence dimensions of subject 1, respectively.

**Table 1:** **Personal threshold for discretizing subjective rating data of arousal and valence dimensions**.

**Subject index**	**Arousal**	**Valence**	**Subject index**	**Arousal**	**Valence**
1	5.6803	5.2342	17	5.1932	5.0815
2	5.6126	6.0166	18	5.5781	5.5596
3	3.7776	5.5513	19	5.4990	5.3685
4	4.5916	4.6503	20	5.6172	5.8185
5	5.1736	4.9791	21	6.0432	5.6618
6	4.6612	5.7579	22	5.3251	4.2624
7	5.0705	4.8358	23	3.6487	6.1354
8	5.6286	5.8466	24	5.8675	4.9634
9	5.6759	5.4592	25	5.9870	5.3552
10	5.0015	5.5064	26	3.8795	4.8234
11	5.1886	4.0322	27	4.6934	5.8161
12	6.3644	4.9731	28	4.7856	5.3817
13	6.6635	4.8578	29	4.3479	4.5732
14	5.4360	4.9597	30	5.1283	5.5714
15	4.7245	5.8538	31	5.6703	4.6661
16	4.7233	4.2413	32	5.6419	5.1586
Mean	5.2012	5.2111			

Based on the target emotion classes defined by the threshold in Table 1, the classification performances shown in the following sections are evaluated by the following metrics. The correct classification rate of low arousal or valence class is,

(3) $$\begin{array}{r} P_{sen}=n_{TP}/\left(n_{TP}+n_{FN}\right), \end{array}$$

with *n*_*TP*_ and *n*_*FN*_ denoting the numbers of correct or incorrect classified low-class instances. The classification accuracy of high arousal or valence class is,

(4) $$\begin{array}{r} P_{spe}=n_{TN}/\left(n_{TN}+n_{FP}\right), \end{array}$$

with *n*_*TN*_ denoting the number of correctively predicted high-level instances and *n*_*FP*_ denoting the number of misclassified high-level instances. The precision for recognizing the low-class instances is defined as *P*_*pre*_,

(5) $$\begin{array}{r} P_{pre}=n_{TP}/\left(n_{TP}+n_{FP}\right), \end{array}$$

The overall classification accuracy is,

(6) $$\begin{array}{r} P_{acc}=\left(n_{TN}+n_{TP}\right)/\left(n_{TN}+n_{FN}+n_{TP}+n_{FP}\right). \end{array}$$

We also employ *F*1-score of low emotion class considering the class imbalance,

(7) $$\begin{array}{r} P_{f}=2P_{pre}P_{sen}/\left(P_{pre}+P_{sen}\right). \end{array}$$

For all equations above, the abbreviations of SEN, SPE, PRE, ACC, TP, TN, FP, and FN denote the Sensitivity, Specificity, Precision, Accuracy, True Positive, True Negative, False Positive, and False Negative, respectively.

### Transfer recursive feature elimination

The standard RFE algorithm is based on a binary SVM classifier. To reduce such high computational cost, T-RFE algorithm is implemented by least square support vector machine (LSSVM), which is known as a SVM variant possessing fast training speed (Suykens and Vandewalle, 1999). Moreover, the linear LSSVM based EEG feature selection and classification approach in our previous work has shown better performance than its nonlinear form due to the low risk of overfitting (Yin and Zhang, 2014). Hence, in this study we employ linear LSSVM via the following optimization problem with constraints of linear equality,

(8) $$\begin{array}{r} \underset{\text{w},b,\xi_{i}}{\min}Z\left(\text{w},b,\xi_{i}\right)=\frac{1}{2}{\text{w}}^{T}\text{w}+\frac{1}{2}\gamma \overset{n}{\underset{i=1}{\sum }}\xi_{i}\\ s.t.\;y_{i}\cdot \left(\text{w}\cdot {\text{x}}_{i}+b\right)=1-\xi_{i},i=1,2,\ldots ,n. \end{array}$$

**Figure 5:**
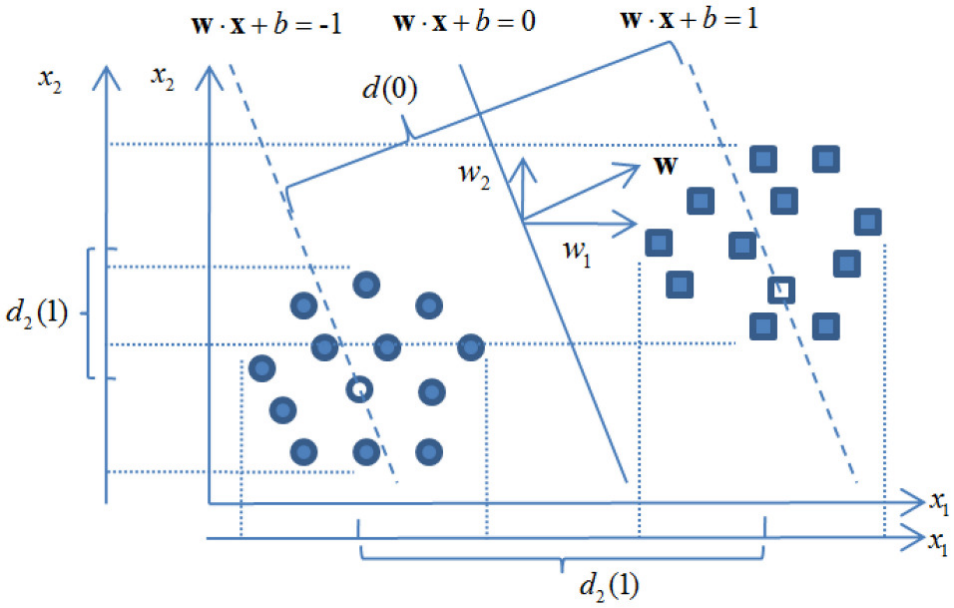
**Least square support vector machine based feature recursive elimination**.

In Equation (8), **w** is the weight vector of the classification hyperplane that classifies *n* training instances **x**_*i*_ (i.e., EEG feature vectors with ${\text{x}}_{i}\in R^{440}$). The distance between the centerline *y*_*i*_ · (**w**·**x**_*i*_ + *b*) = 1 of each class *y*_*i*_ ∈ {−1, 1} is associated with a slack variable ξ_*i*_. A hyper-parameter γ is employed for balancing the penalty term $\overset{n}{\underset{i=1}{\sum }}\xi_{i}$ and the regularization term **w**^*T*^**w**. An example of a LSSVM classification hyperplane and corresponding class centerlines are shown in Figure 5.

The solution of Equation (9) can be derived via Karush-Kuhn-Tucker condition and rearranged as a linear equation system, i.e.,

(9) $$\begin{array}{r} \left[\begin{array}{cc} 0 & -{\text{Y}}^{T}\\ \text{Y}\; & \Psi \Psi^{T}+\gamma^{-1}\text{I} \end{array}\right]\left[\begin{array}{c} b\\ \alpha \end{array}\right]=\left[\begin{array}{c} 0\\ 1 \end{array}\right]. \end{array}$$

In Equation (9), $\text{Y}={\left[y_{1},y_{2},\ldots ,y_{n}\right]}^{T}$, $\alpha ={\left[\alpha_{1},\alpha_{2},\ldots ,\alpha_{n}\right]}^{T}$, and $\text{Z}={\left[{\text{x}}_{1}^{T}y_{1},{\text{x}}_{2}^{T}y_{2},\ldots ,{\text{x}}_{n}^{T}y_{n}\right]}^{T}$ are defined, where α_*i*_, **1**, and **I** denote Lagrangian multiplier for each training instance, a vector with all entries equal to 1 and a 440 × 440 identity matrix, respectively. Given a novel testing EEG feature vector **x**, the estimated emotional class ỹ can be computed by,

(10) $$\begin{array}{r} ỹ=\text{sign}\left(\text{w}\cdot \text{x}+b\right)=\text{sign}\left(\overset{n}{\underset{i=1}{\sum }}\alpha_{i}y_{i}K\left({\text{x}}_{i},\text{x}\right)+b\right), \end{array}$$

with *K*(**x**_*i*_, **x**) = **x**_*i*_ · **x** denoting linear kernel function.

The mechanism of RFE has been also shown in Figure 5. Given a LSSVM classifier is trained by all 440 EEG features, a largest margin of the binary classes *d*(0) can be quantified. Note, that *d*(0) is associated with the regularization term **w**^*T*^**w** while the latter can be computed via,

(11) $$\begin{array}{r} {\left||\text{w}|\right|}^{2}=\alpha^{T}\Psi \Psi^{T}\alpha =\overset{n}{\underset{i=1}{\sum }}\overset{n}{\underset{j=1}{\sum }}\alpha_{i}\alpha_{j}y_{i}y_{j}{\text{x}}_{i}{\text{x}}_{j}. \end{array}$$

When the *kth* feature is eliminated from the feature set, the loss of the classification margin ΔΦ can be measured by,

(12) $$\begin{array}{r} \Delta \Phi =|{\left||\text{w}|\right|}^{2}-{\left||\text{w}\left(k\right)|\right|}^{2}|={\left||w\left(k\right)|\right|}^{2}, \end{array}$$

where **w**(*k*) is the weight vector of the classification plane with *kth* feature eliminated and *w*(*k*) is the *kth* component of **w**. As shown in Figure 5 for a 2-dimensional case, the loss of the margin *d*(0)−*d*_2_(1) with *x*_2_ eliminated is much smaller than *d*(0) − *d*_1_(1) with *x*_1_ eliminated. This observation is consistent with that of *w*_1_ > *w*_2_ and indicates *x*_1_ is a more salient feature since the classification margin (potential generalization capacity) has been much reduced with this feature removed.

**Figure 6:**
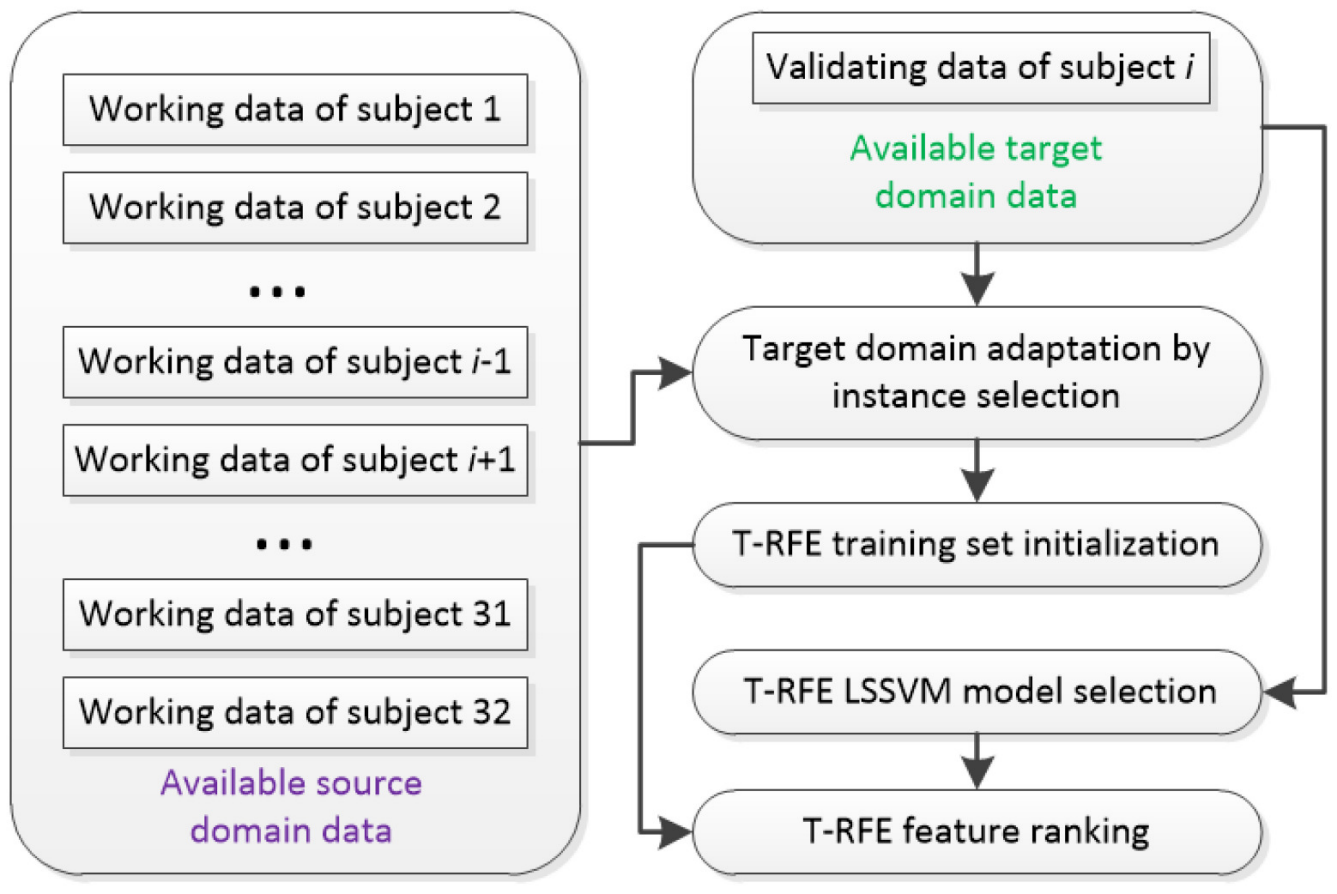
**Initialization of T-RFE training set and LSSVM model**.

Considering the size of subject pool of DEAP is sufficiently large for extracting the shared EEG features across multiple individuals, we define training subjects and a specific testing subject as source domain and target domain, respectively. Then, the scheme for initializing the training set and the LSSVM model for T-RFE can be organized in Figure 6. Given a testing subject *i*, the labeled working EEG data of 31 training subjects that build the source domain are available. For the target domain, the labeled validating data are available while the emotion classes of working data for the testing subject are unknown and required to be predicted.

In order to achieve the source-target domain adaptation, we compute the centers {**v**_*N*_, **v**_*P*_} of low and high emotional states for available target domain data as follows,

(13) $$\begin{array}{r} {{\text{v}}_{P}}^{\left(i\right)}=\frac{1}{n_{P}}\overset{n_{P}}{\underset{j=1}{\sum }}{\text{x}}_{j},{{\text{v}}_{N}}^{\left(i\right)}=\frac{1}{n_{N}}\overset{n_{N}}{\underset{k=1}{\sum }}{\text{x}}_{k},{\text{x}}_{j}\in {V_{P}}^{\left(i\right)},{\text{x}}_{k}\in {V_{N}}^{\left(i\right)}\\ \text{s}.\text{t}.{V_{P}}^{\left(i\right)}\cup {V_{N}}^{\left(i\right)}=V^{\left(i\right)},{V_{P}}^{\left(i\right)}\cap {V_{N}}^{\left(i\right)}=\emptyset . \end{array}$$

In Equation (13), *n*_*P*_ and *n*_*N*_ are the instance numbers of low and high emotion classes, respectively. All instances are from the validating set *V* with *V*_*P*_ denoting low-class subset and *V*_*N*_ denoting high-class subset. Then, the derived {**v**_*N*_, **v**_*P*_} can be used as a reference for manipulating domain adaptation via instance selection since the EEG feature distribution of the available source domain data from the training subjects are different from that of the testing subject. Given a testing subject *i*, the instance selection for source domain data of low emotional state is performed via the following criterion,

(14) $$\begin{array}{r} H_{P}=\frac{N_{{O_{P}}^{\left(i\right)}}||{\text{x}}_{j}-{{\text{v}}_{P}}^{\left(i\right)}||-\overset{N_{{O_{P}}^{\left(i\right)}}}{\underset{j=1}{\sum }}||{\text{x}}_{j}-{{\text{v}}_{P}}^{\left(i\right)}||}{\sqrt{\overset{N_{{O_{P}}^{\left(i\right)}}}{\underset{j=1}{\sum }}\left(N_{{O_{P}}^{\left(i\right)}}||{\text{x}}_{j}-{{\text{v}}_{P}}^{\left(i\right)}||-\overset{N_{{O_{P}}^{\left(i\right)}}}{\underset{j=1}{\sum }}||{\text{x}}_{j}-{{\text{v}}_{P}}^{\left(i\right)}||\right)}}<0,\\ {\text{x}}_{j}\in {O_{P}}^{\left(i\right)},{O_{P}}^{\left(i\right)}\cup {O_{N}}^{\left(i\right)}=O^{\left(i\right)},\\ {O_{P}}^{\left(i\right)}\cap {O_{N}}^{\left(i\right)}=\emptyset ,O^{\left(i\right)}=\underset{l\neq i}{⋃}o^{\left(l\right)}. \end{array}$$

In Equation (14), ${\text{x}}_{j}\in {O_{P}}^{\left(i\right)}$ denotes an EEG feature vector belonging to the low-class subset ${O_{P}}^{\left(i\right)}$ from overall working dataset *O*^(*i*)^, *O*^(*i*)^ is built by the union of all working data *o*^(*l*)^ of subject *l* with *l* ≠ *i*, and $N_{{O_{P}}^{\left(i\right)}}$ is the cardinal number of ${O_{P}}^{\left(i\right)}$. In the end, *H*_*P*_ < 0 indicates the Euclidean distance $\left||{\text{x}}_{j}-{{\text{v}}_{P}}^{\left(i\right)}|\right|$ between the available source and target data is sufficiently small to construct the T-RFE training set. Similarly, the *H*_*N*_ < 0 can be defined to eliminate instances that are far away from the high-class center ${{\text{v}}_{N}}^{\left(i\right)}$. By incorporating the target domain data for selecting LSSVM model, the algorithm for T-RFE initialization is shown in Table 2, where *n*_*s*_ denotes the number of the subjects in DEAP database, $N_{O^{\left(i\right)}}$ is the number of the instances from the source domain, γ_*o*_, Õ^(*i*)^, and *A*^(*i*)^ denote the optimal regularization hyper-parameter, the adaptive source domain set, the initialized training set for T-RFE, respectively. In Table 2, the scheme of evenly selecting 50% instances denotes the every-other data point is selected. That is, the EEG feature vectors with the indices of 1, 3, 5, …are selected and the rests are remained. Inside the loop of the algorithm, the value of *p* = 15 denotes the cardinal number of the candidate set of 2^(−5+*j*)^. That is, we investigate the classification performance of the regularization parameter across the values of 2^(−4)^, 2^(−3)^, …, 2^10^.

**Table 2:** **Pseudo codes of the algorithm for T-RFE initialization**.

**Start T – RFE initialization** **for** *i*= 1: *n*_s_ Define availible target domain set {**x**_*k*_, *y_k_*} = *V*^(*i*)^ from subject *i* Evenly select 50% intances *V_r_*^(*i*)^ from *V*^(*i*)^, *V_r_*^(*i*)^ ⊆ *V*^(*i*)^ **LSSVM model selection** **for** *j* = 1 : *p* Define $\tilde{Z}(w,b,\xi_{k})=\;1/2\cdot w^{T}w+1/2\cdot \;\left(2^{-5+j}\right)\cdot \;\sum_{k\;=\;1}^{n_{V_{r}{}^{\left(i\right)}}}\xi_{k}$ Train LSSVM $y=G_{j}(x)=\text{sign}(\sum_{k\;=\;1}^{n_{V_{r}{}^{\left(i\right)}}}\alpha_{k}y_{k}x_{k}x+b)\text{ via }Z$ Compute *E*(_p_) = 1/2 · (*P_acc_* + *P_f_*) based on *V_r_*^(*i*)^, *G_j_* **End for** **Selected regularization parameter** *γ*_o_^(*i*)^ = 2^−5 + arg min *E*(*p*)^ **Domain adaptation using instance selection** Compute **v***_P_*^(*i*)^, **v***_N_*^*(i)*^ Build availible source domain set *O*^(*i*)^ and subsets *O_P_*^(*i*)^, *O_N_*^(*i*)^ Initialize adaptive source domain set ${\tilde{O}}_{P}^{\left(i\right)}={\tilde{O}}_{N}^{\left(i\right)}=\emptyset$ **for** *j* = 1 : *N_O_*^(*i*)^ Compute $H_{P}(x_{j}),\;x_{j}\in O_{P}{}^{\left(i\right)},or\;H_{N}(x_{j}),\;x_{j}\in O_{N}{}^{\left(i\right)}$ **if** $H_{P}(x_{j})<0\;or\;H_{N}(x_{j})<0$ ${\tilde{O}}_{P}^{{}^{\left(i\right)}}={\tilde{O}}_{P}^{{}^{\left(i\right)}}\cup x_{j}\text{, }or\;{\tilde{O}}_{N}^{{}^{\left(i\right)}}={\tilde{O}}_{N}^{{}^{\left(i\right)}}\cup x_{j}$ **else** ${\tilde{O}}_{P}^{\left(i\right)}={\tilde{O}}_{P}^{\left(i\right)},\;or\;{\tilde{O}}_{N}^{\left(i\right)}={\tilde{O}}_{N}^{\left(i\right)}$ **End if** **End for** ${\tilde{O}}^{\left(i\right)}={\tilde{O}}_{P}^{\left(i\right)}\cup \;{\tilde{O}}_{N}^{\left(i\right)},\;A^{\left(i\right)}=V^{\left(i\right)}\cup {\tilde{O}}^{\left(i\right)}$ **End for**, **Return** γ_o_^(*i*)^, *A*^(*i*)^ **End T – RFE initialization**

The objective function $\Delta \tilde{\Phi }$ of the T-RFE incorporating the difference between the target and source domain can be formularized as the linear combination of two different terms, i.e., the losses of the classification margin for all domains and the reduction of the geometrical distance between source and the target domain for each emotional class,

(15) $$\begin{array}{r} \Delta \tilde{\Phi }=\lambda_{1}\kappa_{1}\left[{\left||w\left(k\right)|\right|}^{2}\right]+\lambda_{2}\kappa_{2}\left\{\left[d_{P}\left(k\right)+d_{N}\left(k\right)\right]\right\}. \end{array}$$

In Equation (15), the term for the margin loss ||*w*(*k*)||^2^ can be computed by Equation (12). It can be also replaced by simply using |*w*(*k*)|. Moreover, *d*_*P*_(*k*)+*d*_*N*_(*k*) is the term for quantifying the distance between the target domain and the source domain. For testing subject *i*, *d*_*P*_(*k*) and *d*_*N*_(*k*) can be computed by,

(16) $$\begin{array}{r} d_{P}\left(k\right)=||{{\text{v}}_{P}}^{\left(i\right)}\left(k\right)-\frac{\text{1}}{N_{{Õ}_{P}^{\left(i\right)}}}\overset{N_{{Õ}_{P}^{\left(i\right)}}}{\underset{j=1}{\sum }}{\text{x}}_{j}\left(k\right)||,{\text{x}}_{j}\in {Õ}_{P}^{\left(i\right)}. \end{array}$$

and,

(17) $$\begin{array}{r} d_{N}\left(k\right)=||{{\text{v}}_{N}}^{\left(i\right)}\left(k\right)-\frac{\text{1}}{N_{{Õ}_{N}^{\left(i\right)}}}\overset{N_{{Õ}_{N}^{\left(i\right)}}}{\underset{j=1}{\sum }}{\text{x}}_{j}\left(k\right)||,{\text{x}}_{j}\in {Õ}_{N}^{\left(i\right)}. \end{array}$$

In Equations (16, 17), the low and high emotion classes are labeled as *P* and *N*, respectively. The centers of the target domain ${{\text{v}}_{P}}^{\left(i\right)}$ and ${{\text{v}}_{N}}^{\left(i\right)}$ can be computed via Equation (14), where (*k*) denotes the *k*^*th*^ feature has been eliminated from the feature set. Note that the subsets for the adaptive source domain ${Õ}_{P}^{\left(i\right)}$ and ${Õ}_{N}^{\left(i\right)}$ are obtained from the T-RFE initialization algorithm listed in Table 2. In addition, the function of κ_1_ and κ_2_ are used to scale the two terms as,

(18) $$\tilde{w}(k)=\kappa [{\left\|w(k)\right\|}^{2}]=\frac{D\;\cdot \;{\left\|w(k)\right\|}^{2}-\sum_{k=1}^{D}{\left\|w(k)\right\|}^{2}}{\sqrt{D\cdot \sum_{k=1}^{D}({\left\|w(k)\right\|}^{2}-\frac{1}{D}\sum_{k=1}^{D}{\left\|w(k)\right\|}^{2}{)}^{2}}},$$

and,

(19) $$\begin{array}{l} \tilde{D}(k)=\kappa [d_{P}(k)+d_{N}(k)]\\ =\frac{D\cdot [d_{P}(k)+d_{N}(k)]-\sum_{k=1}^{D}\left[d_{P}(k)+d_{N}(k)\right]}{\sqrt{D\cdot \sum_{k=1}^{D}\{[d_{P}(k)+d_{N}(k)]-\frac{1}{D}\sum_{k=1}^{D}[d_{P}(k)+d_{N}(k)]{\}}^{2}}}. \end{array}$$

In Equations (18, 19), *D* = 440 is the dimensionality of the initial feature set. Finally, the scaled two terms $\tilde{w}\left(k\right)$ and $\tilde{D}\left(k\right)$ can be weighted and used by using λ_1_ and λ_2_. In this work, we simply employ λ_1_ = λ_2_ = 0.5. That is, the weights between the margin loss of LSSVM and the distance difference of source-target domains are the same. From Equations (15) to (19), the classical RFE has been generalized to T-RFE via a modified evaluation of the feature importance. From ΔΦ to $\Delta \tilde{\Phi }$, the principle of the transfer learning has been suitably incorporated. The algorithm of the T-RFE feature ranking is listed in Table 3, where *q* is the step length that indicates *q* features are eliminated for each iteration. Finally, the algorithm returns the ranked feature set Ẽ while the higher ranking implies the corresponding feature is more salient for cross-subject emotion recognition.

**Table 3:** **Pseudo codes of the algorithm for T-RFE feature ranking**.

**Start T − RFE feature ranking** **for** *i* = 1 : *n_s_* Load target domain set *V*^(*i*)^ from subject *i* Load adaptive souce subsets ${\tilde{O}}_{P}^{\left(i\right)},{\tilde{O}}_{N}^{\left(i\right)}$ Load T-RFE training set {**x**_*k*_, *y*_*k*_} ∈ *A*^(*i*)^, *k* = 1, 2, …,*n*_*A*^(*i*)^_ Load optimal regularization parameter γ_*o*_^(*i*)^ **for** *j* = 1 : *D*/*q* Define $\tilde{Z}(w,b,\xi_{k})=1/2\cdot w^{T}w\;+\;1/2\cdot \;\gamma_{o}{}^{\left(i\right)}\cdot \;\sum_{k\;=\;1}^{n_{A^{\left(i\right)}}}\xi_{k}$ Get Lagrangian $L(w,b,\alpha_{i},e_{i})\;=\;1/2\cdot w^{T}w+...$ $\;...1/2\cdot \gamma_{o}{}^{\left(i\right)}\sum_{k\;=\;1}^{n_{A^{\left(i\right)}}}\xi_{k}-\sum_{k\;=\;1}^{n_{A^{\left(i\right)}}}\alpha_{k}[y_{k}\cdot ((w\cdot x_{i})\;+\;b)\;+\;\xi_{k}-1],$ Get the optimal $\alpha_{k}$ Compute $w=\sum_{k\;=\;1}^{n_{A^{\left(i\right)}}}\alpha_{k}y_{k}\;x_{k}$ **for** $z_{1}=1:D$ $\;\tilde{w}(z_{1})=\kappa \;[{\left\|w(z_{1})\right\|}^{2}]$ Compute $d_{P}(z_{1})\;+\;d_{N}(z_{1}),\tilde{D}(z_{1})\;=\;\kappa [d_{P}(z_{1})\;+\;d_{N}(z_{1})]$ $\;\Delta \tilde{\Phi }\;(z_{1})\;=\;\lambda_{1}\tilde{w}(z_{1})\;+\lambda_{2}\tilde{D}(z_{1})$ **End for** Build feature set *E*(*j*) = ∅ for elimination **for** $z_{2}\;=\;1:q$ $\;E(j)\;=\;E(j)\;\cup \;\arg\;\min\Delta \tilde{\Phi }$ **End for** Eliminate feature set *E* from ${\tilde{O}}_{P}^{\left(i\right)},{\tilde{O}}_{N}^{\left(i\right)},A^{\left(i\right)},V^{\left(i\right)}$ **End for** Get the ranked feature set $\tilde{E}\;=\;\overset{D/q}{\underset{J\;=\;1}{\cup }}E(J)$ **End for**, **Return** $\tilde{E}$ **End T − RFE feature ranking**

## Results

Based on the 32 channel EEG signals from DEAP database, the binary valence and arousal states are estimated by using different feature selection schemes and the linear LSSVM classifier. In total, three different feature selection schemes are employed. (1) Scheme 1, the classical RFE feature ranking is applied to 440 EEG features. The feature ranking is computed via a subject-specific manner. That is, the validating data for each subject is separately used to compute the feature ranking (denoted as RFE-SS). The evaluation of classification performance of RFE-SS is based on the 10-fold cross-validation technique. (2) Scheme 2, the classical RFE algorithm is applied on the feature set and the feature ranking is computed via a cross-subject manner. For a given subject *i* for testing, the ranking is derived from the working segments of all remain 31 subjects (denoted as RFE-SG). Note that the validating data are not used in such case. (3) Scheme 3, the RFE ranking is derived based on the T-RFE algorithm presented in Tables 1, 2 (denoted as TRFE-SG) via the same cross-subject manner. For both of the Schemes 2 and 3, the working segments from the source domain are used for training classifiers while that from the target domain is used for testing and eliciting the performance metrics.

**Figure 7:**
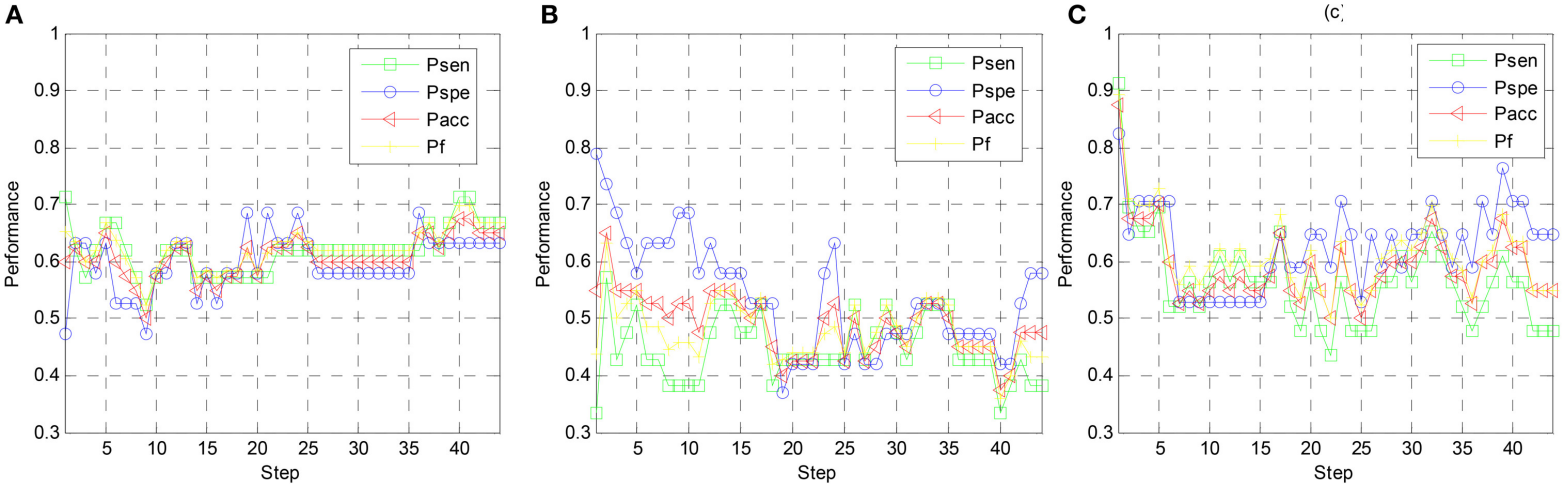
**Arousal classification performance vs. step index of feature elimination for different feature ranking schemes, (A)** RFE-SS, **(B)** RFE-SG, **(C)** TRFE-SG.

To find the optimal number of features that is adopted for emotion classification, the performance metrics are examined on different step indices. Note that the step lengths of all three schemes are set to 10. Namely, for each iteration, 10 EEG features with highest rankings are moved to the feature set. When achieving 44 steps, all 440 EEG features are adopted for emotion classification. In Figure 7, the variation of the arousal classification metrics are explored for subject 1. Four metrics, i.e., *P*_*sen*_, *P*_*spe*_, *P*_*acc*_, and *P*_*f*_, are computed by using the linear LSSVM classifier. For Scheme 1 (RFE-SS) shown in Figure 7A, the optimal *P*_*acc*_ and *P*_*f*_ are derived when 40th step arrives. Since 10 EEG features are iteratively added to the feature set in each step, 40^*^10 = 400 EEG features are required to achieve the optimal classification performance when the classical RFE is subject-specifically implemented. On the other hand, the optimal *P*_*acc*_ and *P*_*f*_ for Scheme 2 (RFE-SG) are derived at 2nd step. It shows that only 20 EEG features are sufficient to achieve the best arousal classification performance when EEG data from multiple training subjects different from the testing subject are used for feature ranking. In particular, the optimal *P*_*acc*_ and *P*_*f*_ for Scheme 3 (TRFE-SG) is found the 1st step while 10 EEG features with the highest rankings elicit best performance. It indicates that the scheme of TRFE-SG can lead to a minimum number of EEG features for estimating arousal states of subject 1. We can also found both values of *P*_*acc*_ and *P*_*f*_ are higher than other two schemes.

**Figure 8:**
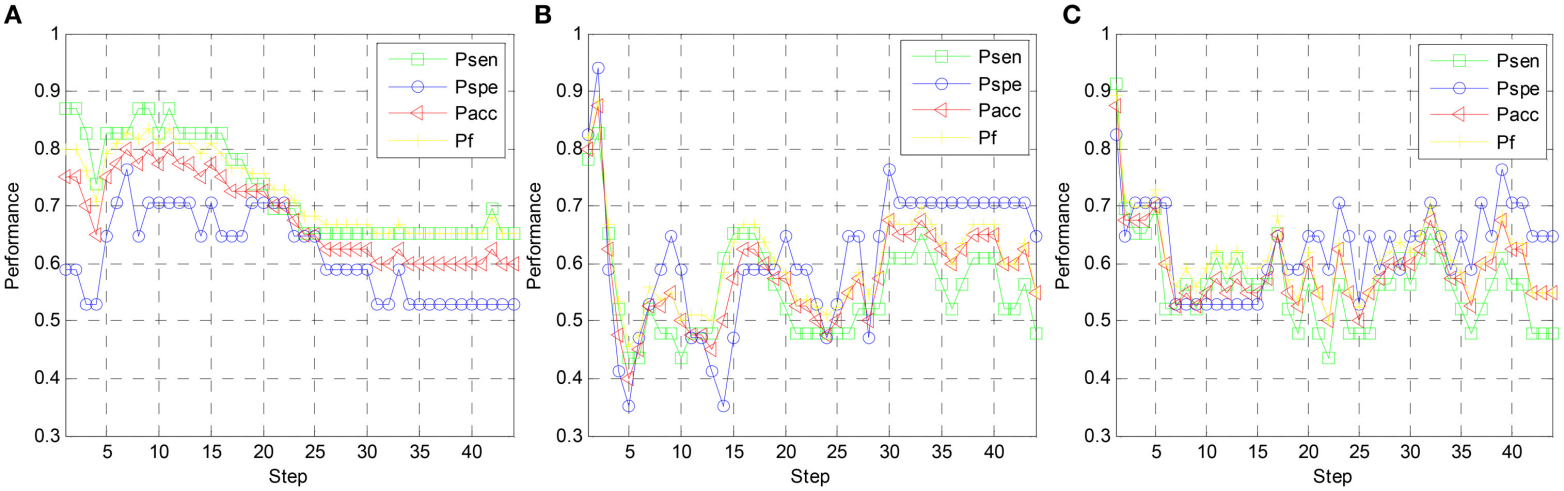
**Valence classification performance vs. step index of feature elimination for different feature ranking schemes, (A)** RFE-SS, **(B)** RFE-SG, **(C)** TRFE-SG.

In Figure 8, the variation of the valence classification performance along with the step index of subject 1 is shown. For Scheme 1, 2, and 3, the optimal *P*_*acc*_ and *P*_*f*_ values are achieved at the 14th step, the 2nd step, and the 1st step, respectively. It indicates for subject-specific RFE, cross-subject RFE, and cross-subject T-RFE, 140, 20, and 10 optimal EEG features are required to get the optimal classification performance. Note that the performance of Scheme 3 is still comparable against the Scheme 2. Regarding the optimal values of *P*_*acc*_ and *P*_*f*_, both of Schemes 2 and 3 are higher than Scheme 1. By observing Figures 7, 8, the high number of salient EEG features are found when feature selection is applied in a subject-specific manner. However, most of the EEG features are redundant or less important for cross-subject emotion recognition and can undermine the generalization capability of the classifier.

To comprehensively evaluate the effectiveness of the TRFE-SG scheme, we build five different emotion classifiers based on linear LSSVM for all 32 subjects. For all classifiers, the regularization parameter is optimized according to the algorithm shown in Table 2. The optimal classification performance is derived for each subject by locating the best step index. The procedure for determining the optimal number of EEG features is as same as those shown in Figures 7, 8. The baseline condition is denoted as LSSVM-SS, where the subject-specific LSSVM is employed to recognize the binary emotional states without any feature selection schemes. The emotion classifier of RFE-LSSVM-SS is defined when only classical RFE feature selection is combined with linear LSSVM in subject-specific manner. Note that the 10-fold cross validation technique is applied to compute classification performance metrics for LSSVM-SS and RFE-LSSVM-SS. Moreover, LSSVM-SG denotes the linear LSSVM is implemented in a cross-subject manner without any feature selection scheme. That is, when a testing subject *i* is given, the remaining 31 subjects are used for training the classifier. Similarly, RFE-LSSVM-SG denotes the classical RFE is applied with LSSVM-SG classifier. Finally, TRFE-LSSVM-SG denotes the T-RFE algorithm is combined with LSSVM-SG classifier.

**Figure 9:**
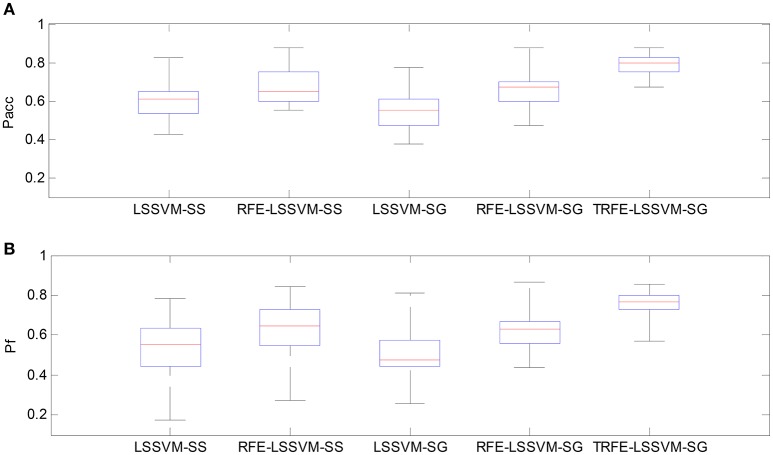
**Box-plots for comparing arousal classification performance across different emotion classifiers for all 32 subjects. (A)** Classification accuracy, **(B)** F1-score.

**Figure 10:**
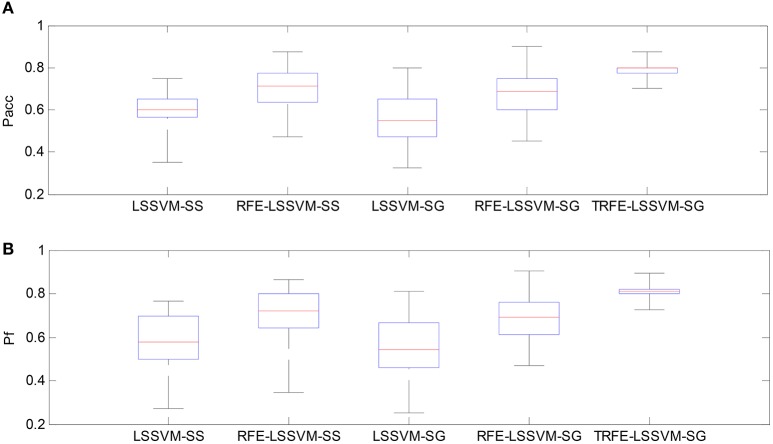
**Box-plots for comparing valence classification performance. (A)** Classification accuracy, **(B)** F1-score.

As shown in Figures 9, 10, the TRFE-LSSVM-SG achieves the best performance regarding *P*_*acc*_ and *P*_*f*_ for predicting both of the arousal and valence dimensions. More specifically, the highest medians are found with the smallest range across all 32 subjects when the T-RFE feature ranking and cross-subject emotion classifier are simultaneously applied. One interesting observation is that the LSSVM-SG achieves the lowest performance. Both of the performance of the LSSVM-SG and the RFE-LSSVM-SG are lower than that of LSSVM-SS and LSSVM-SG. The statistical comparison using the ANOVA test is carried out. The classification performance data are merged into five groups based on five emotion classifiers. The one-way ANOVA is performed and the mean values of *P*_*acc*_ and *P*_*f*_ for both of the arousal and the valence dimensions are significantly varied with *p* < 0.001. Then the multiple comparisons test is applied between each two groups and the corresponding *p* values are shown in Table 4. From the table, the mean values of *P*_*acc*_ and *P*_*f*_ of TRFE-LSSVM-SG classifier for all dimensions are significantly superior to that of LSSVM-SS, RFE-LSSVM-SS, LSSVM-SG, and RFE-LSSVM-SG classifiers with *p* < 0.05.

**Table 4:** **Results of multiple comparison tests using ANOVA for the five emotion classifiers**.

	**LSSVM-SS**	**RFE-LSSVM-SS**	**LSSVM-SG**	**RFE-LSSVM-SG**	**TRFE-LSSVM-SG**
**AROUSAL** *P*_*acc*_					
LSSVM-SS	–	*p* < 0.05	–	*p* < 0.05	*p* < 0.05
RFE-LSSVM-SS	*p* < 0.05	–	*p* < 0.05	–	*p* < 0.05
LSSVM-SG	–	*p* < 0.05	–	*p* < 0.05	*p* < 0.05
RFE-LSSVM-SG	*p* < 0.05	–	*p* < 0.05	–	*p* < 0.05
TRFE-LSSVM-SG	*p* < 0.05	*p* < 0.05	*p* < 0.05	*p* < 0.05	–
**AROUSAL** *P*_*f*_					
LSSVM-SS	–	*p* < 0.05	–	*p* < 0.05	*p* < 0.05
RFE-LSSVM-SS	*p* < 0.05	–	*p* < 0.05	–	*p* < 0.05
LSSVM-SG	–	*p* < 0.05	–	*p* < 0.05	*p* < 0.05
RFE-LSSVM-SG	*p* < 0.05	–	*p* < 0.05	–	*p* < 0.05
TRFE-LSSVM-SG	*p* < 0.05	*p* < 0.05	*p* < 0.05	*p* < 0.05	–
**VALENCE** *P*_*acc*_					
LSSVM-SS	–	*p* < 0.05	–	*p* < 0.05	*p* < 0.05
RFE-LSSVM-SS	*p* < 0.05	–	*p* < 0.05	–	*p* < 0.05
LSSVM-SG	–	*p* < 0.05	–	*p* < 0.05	*p* < 0.05
RFE-LSSVM-SG	*p* < 0.05	–	*p* < 0.05	–	*p* < 0.05
TRFE-LSSVM-SG	*p* < 0.05	*p* < 0.05	*p* < 0.05	*p* < 0.05	–
**VALENCE** *P*_*f*_					
LSSVM-SS	–	*p* < 0.05	–	*p* < 0.05	*p* < 0.05
RFE-LSSVM-SS	*p* < 0.05	–	*p* < 0.05	–	*p* < 0.05
LSSVM-SG	–	*p* < 0.05	–	*p* < 0.05	*p* < 0.05
RFE-LSSVM-SG	*p* < 0.05	–	*p* < 0.05	–	*p* < 0.05
TRFE-LSSVM-SG	*p* < 0.05	*p* < 0.05	*p* < 0.05	*p* < 0.05	–

**Figure 11:**
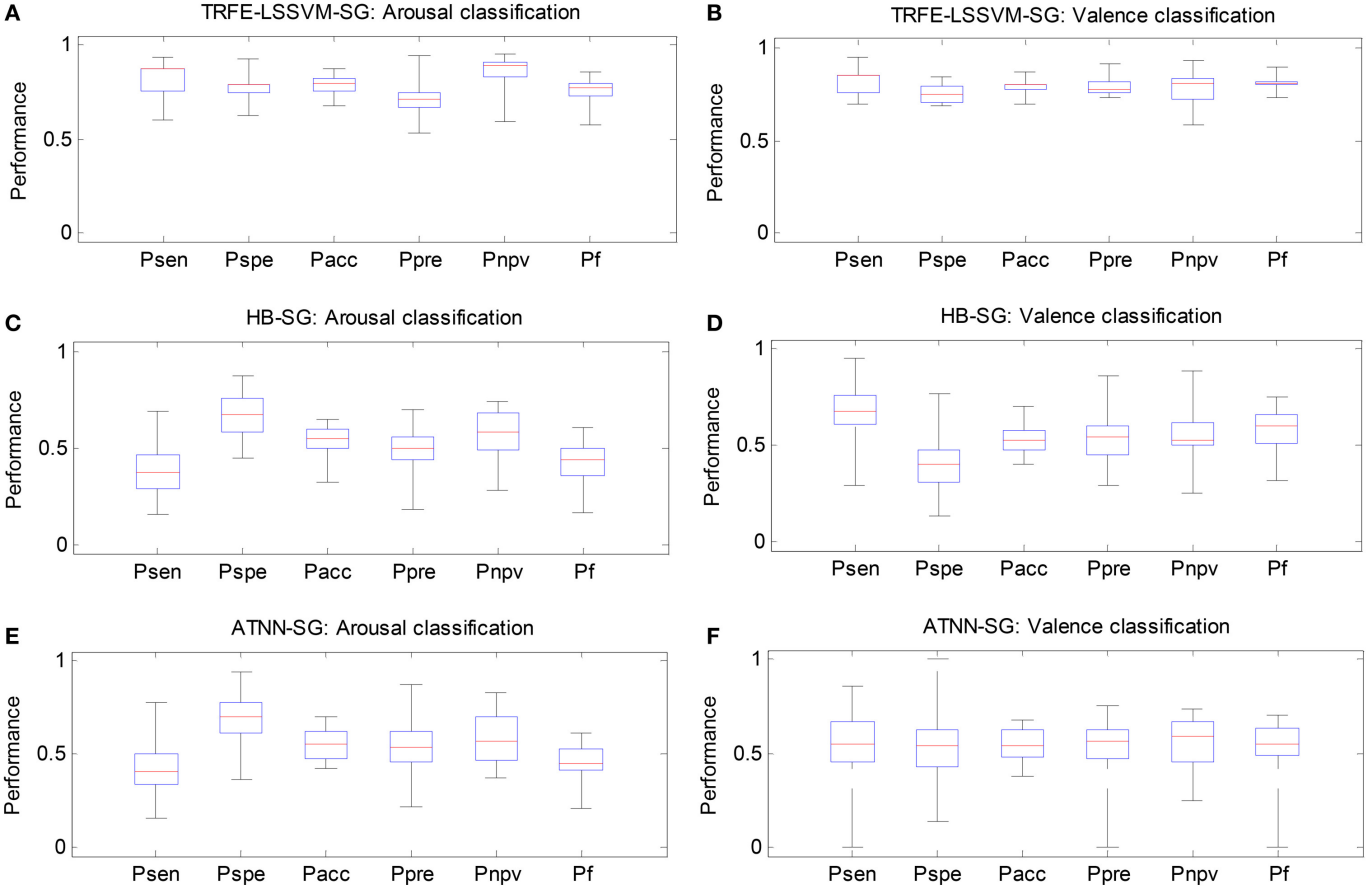
**Classification performance comparison between three subject-generic classifiers. (A,C,E)** Arousal classification results on TRFE-LSSVM-SG, HB-SG, and ATNN-SG. **(B,D,F)** Valence classification results on TRFE-LSSVM-SG, HB-SG, and ATNN-SG.

In Figure 11, we compare the classification performance of the proposed TRFE-LSSVM-SG emotion classifier against two subject-generic classifier, i.e., HB-SG and ATNN-SG. The algorithm of HB-SG is developed based on the work (Wang et al., 2012), where a hierarchical Bayesian (HB) classifier is used for recognizing the change of cognitive states via EEG features across a group of subjects. Here, the hidden variable of the HB classifier is identified by using the principle component analysis. The algorithm of ATNN-SG is developed by following the scheme reported by Baldwin and Penaranda (2012) and an adaptively trained neural network (ATNN) is proposed. For all three classifiers, the classification performance is evaluated by six metrics, i.e., sensitivity, specificity, accuracy, precision, negative-predicting-value and F1-score. The six metrics are denoted by *P*_*sen*_, *P*_*spe*_, *P*_*acc*_, *P*_*pre*_, *P*_*npv*_, and *P*_*f*_, respectively. From the figure, the TRFE-LSSVM-SG classifier achieved the highest value for all metrics. On the other hand, for HB-SG classifier, the sensitivity and specificity are very low for arousal and valence classification, respectively. For ATNN-SG classifier, the range between the lowest and the highest values of each metric is much larger than other cases.

We also compare the subject-average *P*_*acc*_ and *P*_*f*_ values from several reported works for both of the arousal and valence dimensions on the same database in Table 5. In the table, Koelstra et al. (2012) combined the EEG and peripheral features to classify binary emotional classes using SVM and KNN classifier. Liu and Sourina (2012) proposed threshold-based detection algorithm. Naser and Saha (2013) developed the dual-tree wavelet transformation method. Chen et al. (2015) used C4.5 decision tree classifier. Atkinson and Campos (2016) combined the SVM classifier with a mutual information based feature selection approach. Yoon and Chung employed the Bayesian weighted-log-posterior classifier. Li et al. and Wang and Shang adopted deep belief networks for emotion classification. Yin et al. developed an ensemble stacked autoencoder. By comparing the above mention works on the same DEAP database, the TRFE-LSSVM-SG classifier achieves the best classification performance.

**Table 5:** **Subject-average classification performance comparison between TRFE-LSSVM-SG and several reported studies on the DEAP database**.

	**Arousal**		**Valence**	
	***P_*acc*_***	***P_*f*_***	***P_*acc*_***	***P_*f*_***
Koelstra et al., 2012	0.6200	0.6310	0.6270	0.6520
Liu and Sourina, 2012	0.7651	–	0.5080	–
Naser and Saha, 2013	0.6620	–	0.6430	–
Chen et al., 2015	0.6909	0.6896	0.6789	0.6783
Atkinson and Campos, 2016	0.7306	–	0.7314	–
Yoon and Chung, 2013	0.7010	–	0.7090	–
Li et al., 2015	0.6420		0.5840	
Wang and Shang, 2013	0.5120		0.6090	
Yin et al., 2017	0.7719	0.6901	0.7617	0.7243
TRFE-LSSVM-SG	0.7867	0.7526	0.7875	0.8077

In the end, the computational time of the six classifiers is summarized in Table 6. For each classifier, the summation of the training time for all 32 subjects is recorded and the subject-average time is computed for the comparison. The testing time of 40 EEG feature vectors from each subject is derived in the same manner. All codes of the algorithms were written by using Matlab® 2011b and run on a computer with AMD® CPU 2.0GHZ, 8GRAM and Windows 8® operating system. From the table, LSSVM-SS and TRFE-LSSVM-SG achieve the lowest and the highest training time, respectively. The reason is that the T-RFE algorithm requires additional steps to evaluate the distribution difference s between the source and target domains. Hence, the high accuracy of the TRFE-LSSVM-SG is at the cost of the high training time. For the testing time, HB-SG and LSSVM-SG achieve the lowest and the highest value, respectively. Note that for all classifiers the average testing time is smaller than 100 ms, which indicates the trained classifiers could implement online. In such case, the TRFE-LSSVM-SG is very competitive against other classifiers because of the low testing time and high classification performance.

**Table 6:** **Subject-average CPU time (in s) for classifier training and testing**.

	**Training**	**Testing**
LSSVM-SS	0.0625	0.0469
RFE-LSSVM-SS	7.4063	0.0094
LSSVM-SG	1.4688	0.0938
RFE-LSSVM-SG	19.4810	0.0104
HB-SG	0.0996	0.0016
ATNN-SG	9.6406	0.0157
TRFE-LSSVM-SG	80.8736	0.0125

## Discussions

Regarding the methodology, we have generalized a classical SVM-based feature selection algorithm for solving cross-subject emotion classification problem, where the EEG features possess the non-stationarity and differently distributed across multiple individuals. The essential of the proposed T-RFE algorithm is to introduce the transfer learning principle and quantify the difference of high dimensional EEG feature distributions between the source domain and the target domain. The objective function of the T-RFE is a linear combination of the classification margin and the geometrical distance for domain adaptation. In addition, the instance selection has been applied to initialize a rich training set with sufficient training samples. For the subject-specific emotion recognition, the size of the training set of a subject is smaller than the cross-subject paradigm. It is because the EEG data from other subjects are not exploited. By selecting and fusing EEG data from different individuals, a domain adaptable set is used for ranking features, which shares common information for multiple individuals. Note, that the ground truth of each EEG feature vector is predetermined by subject-specific clustering of self-assessment data. An adaptive threshold reflects the preference and the personality for participants rating the musical video clips and is more reasonable than using a fixed threshold.

On the other hand, the classification performances between the subject-specific and cross-subject emotion recognition paradigms are compared. The first observation is that the performance of the LSSVM-SG is lower than that of the LSSVM-SS scheme. Based on the Wilcoxon signed rank test, the significant decrease of the classification accuracy has been found with (*p* = 0.049, *z* = −2.0). Moreover, the classical RFE algorithm has also shown better performance in subject-specific paradigm. However, there is no significant difference of the average classification rate and *F*-score between the RFE-LSSVM-SS and RFE-LSSVM-SG classifiers. It implies that fusing the EEG data from multiple subjects together to build the training set cannot improve the generalization capability. The classical RFE also does not benefit from the sufficient training instances across multiple individuals. The potential reason is the dynamics of EEG feature in other subjects may be quite different from the training subjects and may reduce the classification margin. Finally, the proposed T-RFE has been combined with the linear LSSVM classifier and the significant improvement has been found against all other classifiers. It is also noted that the structure of the linear LSSVM is more transparent than neural network based classifiers and can be constructed by fast training algorithm. The transparency indicates the model structure of the linear LSSVM is much simpler than the NN based classifier. For NN classifier, the non-linear activation function is usually employed and the classification decision function is combined from the hidden activation potentials. That is, the relationship between the input EEG features and the emotional states is modeled via a complex non-linear mapping. On the other hand, the linear LSSVM model can be represented by a linear hyper-plane in the feature space, where the absolute value of its normal vector reflects classification contribution of each EEG feature. We found that the classification performance for both of the arousal and valence dimensions of the TRFE-LSSVM-SG are better than several reported works on the same DEAP database, where very complex classifiers, i.e., deep learning primitives and variants, are applied. It indicates the proper selection of the high dimensional EEG features may play a more important role in a physiological feature based emotion recognition system.

The comparison of the computational complexity indicates the additional training time is required for TRFE-LSSVM-SG classifier. The reason is that the T-RFE algorithm evaluates the loss of the classification margin across the target and source domains. Such operation is performed each iteration and takes much longer time than the conventional RFE algorithm. Note, that the training time of RFE-LSSVM-SG is also higher than that of RFE-LSSVM-SS. It is because the subject-generic classifier is built via a much larger training set. When feature selection is not applied, LSSVM-SS achieves the shortest training time. The reason behind is the LSSVM construction only requires solving a linear equation system for once. The testing time of all classifiers is much less than the training time since all model parameters have been predetermined and only the computation of the outputs is needed. It is also shown the RFE and T-RFE based classifiers possess much less testing time than LSSVM-SS and LSSVM-SG since the dimensionality of the input EEG features has been largely reduced when applying feature selection.

Since both of the historical data from the training and testing subjects are needed to build the T-RFE feature selection model, we construct a validating set by using the first 10 s EEG signals of each trial. When a subject is used for testing, its validating data build the target domain dataset and they are non-overlapped with the testing dataset. On the other hand, if all 60 s data are used for T-RFE modeling as well as evaluating classifier performance, the feature selection procedure exploits the information of the testing dataset and the potential overfitting may arise. In addition, if the validating data are unavailable, the information from the target domain is unknown and the knowledge transfer becomes impossible for the T-RFE algorithm. On the other hand, we also employ the linear SVM based classifier to control the overfitting of the T-RFE. The linear model has a parsimonious structure than the non-linear model and the SVM follows the principle of the structural risk minimization. Both of them are proper to tackle the current classification task with limited instance amount and high feature dimensionality.

There are two important parameters of the T-RFE algorithms, i.e., lamda1 and lamda2, need to be carefully selected. These two parameters stand for the importance of the classification margin and the distance between data distribution of the target and source domains when applying EEG feature elimination. On one hand, the two parameters can be determined based on the prior knowledge. In this study, the two factors are treated with the same importance, i.e., λ_1_ = λ_2_. Since such approach could lead to the suboptimal values, an alternative way is to employ the validating set and candidate parameter set to find the best lambda1 and lambda2 according to the optimal classification performance. Specifically, for each pair of (λ_1_, λ_2_) belongs to {(0.1, 0.9), (0.2, 0.8), …, (0.9, 0.1)} with 10 candidate combinations. The feature selection results and the classification performance on the validating set can be elicited. Then, the optimal values of lambda1 and lambda2 correspond to the highest performance can be determined.

The reason for selecting the hyper-parameter γ is that the LSSVM need to balance the weights between the classification margin and the training error. The best gamma indicates the optimal balance between the two terms above. In this study, the method for the γ optimization also depends on the validating set and a candidate parameter set of {2^−4^, 2^−3^, …, 2^10^} with 15 candidate values. After examining all 15 cases, the optimal classification performance on the validating set yields the best value of γ.

The limitations of this work may cover the following two aspects:

1. The implementation of the proposed T-RFE algorithm requires the emotion class labels of the validating set from the target domain. The reason is that the SVM based RFE feature ranking is naturally a supervised learning approach. The future work should include developing the semi-supervised version of the T-RFE, in which the feature selection procedure can be robust against the unknown label from the target domain.
2. The T-RFE algorithm has lead to several additional hyper-parameters that should be carefully selected, i.e., weight parameter of the T-RFE objective function. In particular, the threshold for instance selection is set to 0 in this study. The cross-validation based model selection may further improve the suitability of the initialized training set. However, such optimization problem can induce additional computational cost.

In our future work, the proposed T-RFE algorithm will be evaluated in the mental workload and mental fatigue recognition tasks, where the multimodal physiological features are used as the cues of the model. It is also possible to generalize the T-RFE method to the cross-session and cross-task operator-functional-state estimation issue aiming at improving the stability of the classification performance. After improving the training speed of T-RFE method in near future, all codes will be optimized into a MATLAB toolbox and available online. Since the EEG signal devices may be intrusive to the users of the human-machine system when collecting emotional clues, the T-RFE based emotion classifier can be evaluated by using videos of the user expressions or recording of the user speech in our future work. To reduce the invasion degree of the EEG sensors, one solution is to use a single-channel, wireless recording devise. The single EEG feature can be combined with the data of the user expression and speech to achieve a multimodal emotion classifier. On the other hand, the emotions can be also linked to the workload and fatigue estimation when the operator is performing a safety critical task. For instance, the anxiety is related to the high workload and may induce the fatigue accumulation. Since the degradation of the human performance may arise due to negative emotions, the employment of the physiological signal is necessary since the cognitive state can be continuously predicted.

## Conclusions

In this study, a new feature selection approach, T-RFE, has been proposed to determine the optimal feature subset regarding a cross subject emotion classification issue. The EEG data from 32 participants in the DEAP database have been employed to examine the effectiveness of the proposed method. Different from the conventional subject-specific paradigm, the training and testing EEG data are coming from different individuals. By properly defining a limited validating set of EEG feature set with 440 dimensions, the objective function of the T-RFE introduces a penalty term that quantifies the difference of feature distributions between the source the target domains. By implementing the linear LSSVM classifier and the non-parametrical statistical test, the significant improvement has been found for the T-RFE feature selection in a cross-subject manner than the cases of conventional RFE methods in both of the subject-specific and subject-generic manners. The overall findings also indicate both of the features and instances should be carefully selected before implementing a cross-subject classifier for non-stationary EEG features.

## Author contributions

ZY developed the T-RFE algorithm, performed all the data analysis, and wrote the manuscript. YW, LL, WZ, and JZ advised data analysis and edited the manuscript.

### Conflict of interest statement

The authors declare that the research was conducted in the absence of any commercial or financial relationships that could be construed as a potential conflict of interest. The reviewer RC and handling Editor declared their shared affiliation, and the handling Editor states that the process nevertheless met the standards of a fair and objective review.
